# Surface-Functionalized NdVO_4_:Gd^3+^ Nanoplates as Active Agents for Near-Infrared-Light-Triggered and Multimodal-Imaging-Guided Photothermal Therapy

**DOI:** 10.3390/pharmaceutics14061217

**Published:** 2022-06-08

**Authors:** Kerong Deng, Donglian Liu, Ziyan Wang, Zhaoru Zhou, Qianyi Chen, Jiamin Luo, Yaru Zhang, Zhiyao Hou, Jun Lin

**Affiliations:** 1 The Sixth Affiliated Hospital of Guangzhou Medical University, Qingyuan People’s Hospital, Qingyuan 511518, China; dengkr26@gmail.com (K.D.); ldl00987@163.com (D.L.); 2 Guangzhou Municipal and Guangdong Provincial Key Labrotory of Protein Modification and Degradation, School of Basic Medical Sciences, Guangzhou Medical University, Guangzhou 511436, China; 2019218158@stu.gzhmu.edu.cn (Z.Z.); 2019218157@stu.gzhmu.edu.cn (Q.C.); jiaminluo7@stu.gzhmu.edu.cn (J.L.); 3 State Key Laboratory of Rare Earth Resource Utilization, Changchun Institute of Applied Chemistry, Chinese Academy of Sciences, Changchun 130022, China; 4 The First Clinical School of Guangzhou Medical University, Guangzhou Medical University, Guangzhou 510120, China; wangzy2018@stu.gzhmu.edu.cn

**Keywords:** near infrared light, NdVO_4_, polydopamine, photothermal therapy, multimodal imaging

## Abstract

Development of nanotheranostic agents with near-infrared (NIR) absorption offers an effective tool for fighting malignant diseases. Lanthanide ion neodymium (Nd^3+^)-based nanomaterials, due to the maximum absorption at around 800 nm and unique optical properties, have caught great attention as potential agents for simultaneous cancer diagnosis and therapy. Herein, we employed an active nanoplatform based on gadolinium-ion-doped NdVO_4_ nanoplates (NdVO_4_:Gd^3+^ NPs) for multiple-imaging-assisted photothermal therapy. These NPs exhibited enhanced NIR absorption and excellent biocompatibility after being grafted with polydopamine (pDA) and bovine serum albumin (BSA) layers on their surface. Upon expose to an 808 nm laser, these resulting NPs were able to trigger hyperthermia rapidly and cause photo-destruction of cancer cells. In a xenograft tumor model, tumor growth was also significantly inhibited by these photothermal agents under NIR laser irradiation. Owing to the multicomponent nanostructures, we demonstrated these nanoagents as being novel contrast agents for in vivo magnetic resonance (MR) imaging, X-ray computed tomography (CT), photoacoustic (PA) imaging, and second biological window fluorescent imaging of tumor models. Thus, we believe that this new kind of nanotherapeutic will benefit the development of emerging nanosystems for biological imaging and cancer therapy.

## 1. Introduction

Although numerous treatment modalities are available for fighting cancer, photothermal therapy (PTT), a minimally noninvasive and highly efficient treatment modality, has still been an active field of research [1,2,3,4,5]. It currently relies on the precise energy delivery to target sites and the sensitivity of tumor area to temperature increase, resulting in irreversible cell damage for cancer ablation [6,7,8]. Considering the nonspecific thermal effects, nanoparticle-assisted PTT seems to be an area of intense interest recently. Taking advantage of laser-induced nanoparticles, it can be easier to localize heat generation inside the desire area to improve the efficacy of PTT and avoid potential damage to surrounding healthy cells [9,10,11,12]. So far, shallow light penetration in living tissue remains a major limitation for in vivo applications of conventional PTT, which has hindered its therapeutic efficiency. Great efforts have been made to develop photothermal conducting nanoparticles active in the near infrared region (700–1400 nm), lying in the “biological window” [13,14,15]. Due to relatively lower scattering and absorption from the intrinsic tissue chromophores, NIR light provides maximal penetration of biological tissue [16,17,18,19,20]. Various inorganic nanoparticles with effective NIR absorption have been studied as photothermal agents for NIR light-mediated PTT [21,22,23,24,25,26], such as gold nanostructures and carbon-based nanostructures, and have achieved effective therapeutic outcomes in vivo. Nevertheless, single functionality for cancer treatment cannot meet the increasing needs for personalized treatment of early diseases. To fabricate photothermal nanoagents with advanced performance through the integration of imaging capability may bring about a great improvement in photothermal ablation therapy due to the real-time monitoring of therapeutic action with more accuracy and efficacy [27].

As a promising candidate for PTT, Nd^3+^-based nanomaterials have attracted much attention recently. With high-absorption cross-section at around 800 nm and multiphonon relaxation of Nd^3+^, these nanoparticles are increasingly attractive for photothermal agents to convert the absorbed radiation into heat as an 808 nm light-triggered nanoheater [28,29,30,31,32]. For example, ultrasmall NdVO_4_ nanoparticles as heating agents to improve photothermal conversion efficiency have been explored [33]. More intriguingly, the main emission bands of Nd^3+^ at around 1050 nm and 1330 nm both lie in the “second biological window” (II-BW), which extends from 1000 nm up to 1400 nm [34]. The optical scattering in the II-BW has been proven to be reduced when compared to that in the I-BW, affording an improvement in resolution of sub-tissue images. Thus, these unique properties of Nd^3+^-based nanomaterials may pave a new way to achieve multifunctional therapeutic agents with deep-tissue imaging capability. Even though multimodal imaging satisfies current requirements for clinical diagnosis, the development of appealing Nd^3+^-based nanoagents for multimodal imaging guided phototherapy is minimally reported to our best knowledge.

**Scheme 1:**
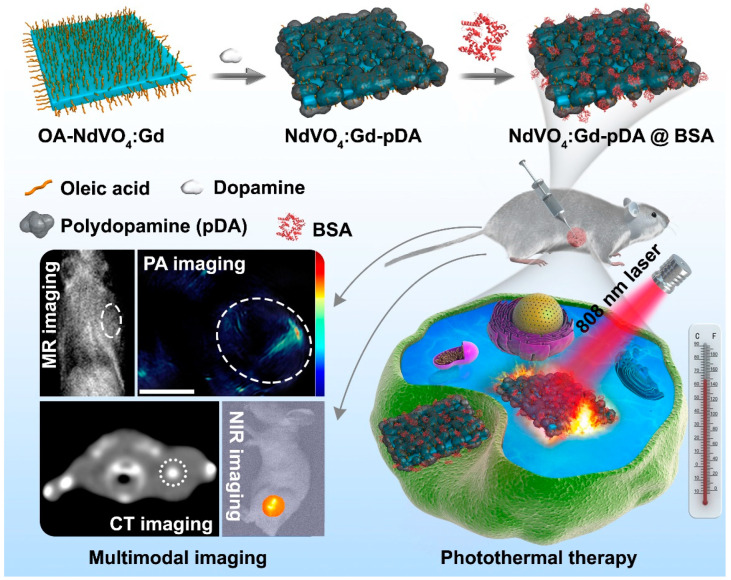
Schematic illustration of the synthesis process of NV-p@BSA nanoplates and their potential bioapplication for in vivo multimodal-imaging-guided photothermal therapy under 808 nm laser irradiation.

In this work, we present a versatile nanoplatform based on unique NdVO_4_:Gd^3+^ NPs for in vivo multimodal imaging and activatable NIR-induced photothermal therapy of cancer. In this design, Nd^3+^-based nanomaterials have a strong absorption at 808 nm and could serve as a therapeutic agent for selective phototherapy using a low-energy NIR laser. The synthetic procedure and potential bioapplication of NdVO_4_:Gd^3+^-pDA@BSA (NV-p@BSA) NPs are illustrated in Scheme 1. Firstly, Gd^3+^-doped NdVO_4_ NPs were synthesized by the one-step method and then modified with polydopamine on their surface. Polydopamine modification not only facilitates the following hydrophilicity but also enhances the photo-to-heat response via its photothermal ability. Next, in order to enhance water-solubility and biological properties of these NPs, negatively charged BSA molecules were chosen as a model biological agent to conjugate on their surface to achieve NV-p@BSA NPs. Both in vitro and in vivo investigations indicated that NV-p@BSA NPs possess excellent biocompatibility and show effective photothermal therapeutic upon NIR irradiation. More importantly, these nanocrystals are capable of emitting luminescent signal around 1050 nm in the second window of the near-infrared (NIR-II) region, which is particularly suitable for deep tissue fluorescence imaging. Meanwhile, after administrated to tumor-bearing mice, these NPs also exhibit the ability of magnetic resonance (MR)/X-ray computed tomography (CT)/photoacoustic (PA)/NIR-II fluorescent multimodal imaging for visualizing the tumor areas. On the basis of the combination of these properties, we demonstrate the novel nanoplatform based on NdVO_4_:Gd^3+^ NPs for multimodal-imaging-guided photothermal therapy.

## 2. Materials and Methods

### 2.1. Chemicals and Instrumentation

Nd(NO_3_)_3_·6H_2_O and Gd(NO_3_)_3_·6H_2_O were purchased from Beijing HWRK Co., LTD (Beijing, China). NH_4_VO_3_ was purchased from Sinopharm Chemical Reagent Co., Ltd. (Shanghai, China). Dopamine hydrochloride and polyethylene glycol (PEG) were acquired from Sigma-Aldrich Co. (Saint Louis, MO, USA). Dimethyl sulfoxide (DMSO) was obtained from Xilong Chemical Co., Ltd. (Swatow, China). Bovine serum albumin (BSA) was obtained from Shanghai Aladdin Biochemical Technology Co., Ltd. (Shanghai, China). All chemicals were used as received.

TEM images of NPs were acquired from a FEI Tecnai G2 S-Twin operating at an acceleration voltage of 200 kV. The X-ray powder diffraction was obtained using a D8 Advanced diffractometer (Bruker) with Cu Kα radiation (λ = 0.15405 nm). The UV–VIS absorption was recorded on U-3100 spectrophotometer (Hitachi). The Zeta potential was measured using a Malvern instrument Zetasizer Nano. The Confocal laser scanning microscopy images was performed on a LSM FV 1000 instrument (Olympus). Thermogravimetry data were recorded on a Netzsch Thermal Analyzer (STA 409) in an ambient environment with a heating rate of 10 °C min^−1^ from room temperature to 700 °C.

### 2.2. Synthesis of Oleic-Acid-Capped NdVO4:Gd^3+^ Nanoplates

Oleic-acid-capped NdVO_4_ NPs were prepared via a facile hydrothermal method [35]. Briefly, 0.5 mmol NH_4_NO_3_ was added into 5 mL water containing 15 mmol NaOH to form a transparent solution. Then, 20 mL of a mixed solution of oleic acid and ethanol (*v*/*v* = 1:1) was added under strong stirring. Subsequently, 1 mL Nd(NO_3_)_3_ aqueous solution (1 mol L^−1^) was added dropwise. After stirring for 10 min, the resulting solution was transferred into a Telfon-lined vessel and sealed, followed by treatment at 140 °C for 8 h. The as-prepared NPs were collected after the autoclave cooled to room temperature naturally. For purification, these NPs were washed with cyclohexane and precipitated by ethanol twice. The 20% Gd^3+^-doped NdVO_4_ NPs were synthesized by the same procedure as described above, except we introduced 0.8 mL Nd(NO_3_)_3_ aqueous solution (1 mol L^−1^) and 0.2 mL Gd(NO_3_)_3_ aqueous solution (1 mol L^−1^) to the precursor mixture.

### 2.3. Synthesis of NdVO_4_:Gd^3+^-pDA@BSA NPs

The modification of NdVO_4_:Gd^3+^ NPs was performed according to a previously published protocol [36]. Briefly, as-obtained NdVO_4_:Gd^3+^ NPs were dispersed in 5 mL chloroform solution. Then, 20 mg dopamine hydrochloride was dissolved into 5 mL DMSO and added into the above solution under stirring to form a homogeneous solution. Subsequently, the mixture was heated to 70 °C for 1 h. After cooling down to room temperature, the nanoparticles were collected via centrifugation and washed with the mixture of DMSO and CHCl_3_ (*v*/*v* = 1:1). Finally, 5 mL DMSO solution of NdVO_4_:Gd^3+^-pDA (20 mg) was slowly added to 10 mL BSA aqueous solution (1 mg mL^−1^) to form a homogeneous solution with sonication. After stirring for 2 h, the mixture was separated by centrifugation and washed by water repeatedly to obtain NdVO_4_:Gd^3+^-pDA@BSA nanoplates. To obtain PEG-attached NdVO_4_:Gd^3+^ nanoplates, a total of 5 mL of a chloroform dispersion of NdVO_4_:Gd^3+^ nanoplates (0.1 mmol) was mixed with a chloroform solution (1 mL) containing 25 mg polyethylene glycol in a round bottom flask. Chloroform was then removed by evaporating slowly under nitrogen atmosphere for 24 h at room temperature. Then, the obtained mixed film was hydrated with deionized water (5 mL) after vigorous sonication. The products were collected by centrifugation at 12,000 rpm for 30 min and rinsed by deionized water at least three times.

### 2.4. In Vitro Photothermal Effects

A total of 200 μL of NdVO_4_:Gd^3+^-pDA aqueous suspension at various concentrations (31.25, 62.5, 125, 250, and 500 μg mL^−1^) was placed in test wells. The solution was irradiated with 808 nm laser (1.0 W cm^−2^, Changchun New Industries Optoelectronics Technology Co., Ltd., Changchun, China) for 5 min, and an infrared camera (FLIRT62101) was utilized to monitor the temperature change during this period. For comparison, the aqueous solution and PEG-modified NdVO_4_:Gd^3+^ suspension was used as control. In this work, the particle concentrations were calculated on the basis of total mass of the resulting nanoplates. To keep the consistent particle concentration, the same calculation method was utilized through all the in vitro and in vivo experiments.

### 2.5. In Vitro Cytotoxicity and Phototoxicity Analyses

The in vitro cytotoxicity was tested with HeLa cells and L929 cells by 3-(4,5-dimethylthia-zol-2-yl)-2,5-diphenyltetrazolium bromide (MTT) assay. The cultured HeLa cells and L929 cells were transferred, at a density of 6000 cells per well, to a 96-well plate and kept at 37 °C for 24 h. Then, NdVO_4_:Gd^3+^-pDA@BSA NP solutions were added to yield the final concentrations of 6.25, 12.5, 25, 50, 100, 200, 250, and 300 μg mL^−1^, and the cells were then incubated with NPs for 24 h. Following this, the supernatant was removed, and 20 μL of MTT was added. After incubation, the medium was replaced with 150 μL of DMSO in each well. Subsequently, the plate was examined using a microplate reader, and cell viability was determined from absorbance at 490 nm relative to the controls.

To evaluate the photothermal cytotoxicity of NPs, HeLa cells were seeded onto 96-well plates for 24 h to adhere, and a series of concentrations of the NPs solution (15, 31, 62.5, 125, 250, and 300 μg mL^−1^) were added for additional 4 h incubation. After that, the cells were exposed to a 808 nm laser for 5 min at a fixed power density of 1.0 W cm^−2^. The cells were incubated for another 24 h. In parallel, the cells without any treatment were exposed to the laser light serving as a negative control. The phototoxicity of NPs was assessed by the MTT viability assay as described above.

### 2.6. Cellular Uptake of NdVO_4_:Gd^3+^-pDA@BSA NPs

HeLa cells were seeded in 6-well plate at a density of 8 × 10^4^ cells per well and cultured overnight. Then, the medium was replaced with fresh culture medium containing NPs (150 μg mL^−1^). After incubation for 4 h, the cells were washed with PBS and fixed with 10% paraformaldehyde. TEM images of fixed cells were taken on a Hitachi H-7650 TEM (Tokyo, Japan). For confocal laser scanning microscopy, FITC were used to label these NPs. Briefly, HeLa cells were incubated with FITC-labeled NPs at 37 °C for 4 h. After medium removal, the cells were washed with PBS and fixed in 4% paraformaldehyde for 10 min. For nucleus labeling, the cells were incubated with DAPI solution for 10 min. Then, the medium was removal and rinsed with PBS several times. The cells were examined with a confocal microscope.

### 2.7. Cell Lines and Animal Tumor Model

The human cervical cancer cells (HeLa), mouse hepatoma cells (H22), and mouse fibroblast cells (L929) were originally obtained from American Type Culture Collection (ATCC). HeLa cells and H22 cells were cultured in RMPI 1640 medium (Gibco) supplemented with 10% fetal bovine serum (Gibco) and 1% penicillin/streptomycin (Invitrogen) at 37 °C in 5% CO_2_. L929 cells were cultured in DMEM medium supplemented with 10% fetal bovine serum (Gibco) and 1% penicillin/streptomycin (Invitrogen) at 37 °C in 5% CO_2_. Healthy female Balb/c mice (six weeks old) were purchased from Guangdong Yaokang Biotechnology Co., Ltd. (Foshan, China). The mouse hepatoma cells H22 tumor model was established by subcutaneous injection with H22 cells (5 × 10^5^ per mouse) into the left flank of healthy Balb/c mice. When the tumor volume reached an approximate volume of 100 mm^3^, the in vivo studies were carried out. All the animal studies were fully compliant with the guidelines of National Institutes of Health Animal Care and Use Committee (NIHACUC) approved protocol.

### 2.8. In Vivo Multimodal Imaging

In vivo multimodal imaging experiments were performed immediately after intratumoral injection. For each imaging model, the time interval between injection and imaging could have been somewhat different due to the time required for image processing. Each imaging modality was separately performed on different mice, since the MR/CT/PA imaging equipment was located differently with restricted user access. For in vitro MR imaging, a series of NV-p@BSA aqueous solutions were prepared. The MR images were taken from a 1.2 T MR scanner for a small animal imaging system (Shanghai Huantong Science and Education Equipment Co., LTD. Shanghai, China). To perform in vivo MR imaging, 200 μL of NPs (20 mg kg^−1^) were intratumorally injected into the anesthetized mice. The MR images were acquired before injection and after injection.

To perform X-ray CT imaging in vivo, tumor-bearing mice were first anesthetized with 10% chloral hydrate by intraperitoneal injection. A total of 100 μL of NV-p@BSA NPs was intratumorally injected into tumor-bearing mice in situ. Then, the mouse was scanned before injection and after injection. CT signal was recorded on a Philips 256-slice CT scanner (Philips Medical System).

For in vivo PA imaging, the mice bearing H22 xenograft tumors were anesthetized with 2% isofluorane and then located in prone position. Photoacoustic signals were detected at a wavelength of 808 nm. The PA images of the tumor area were monitored via a preclinical photoacoustic computed tomography scanner (MSOT InVision128) before and after injection of NPs (20 mg kg^−1^).

For in vivo thermal imaging, 100 μL of saline, NV-PEG, and NV-p@BSA NPs were intratumorally injected into the tumors, individually. After injection, the tumor site was exposed to NIR laser at an output power of 1.0 W cm^−2^, and IR images were recorded using infrared camera.

For in vivo NIR-II fluorescent imaging, 100 μL of NV-p@BSA NPs were intratumorally injected into the tumors. After injection, the tumor was exposed to NIR laser at an output power of 1.0 W cm^−2^, and second biological windows fluorescent imaging was recorded using NIR II Fluorescence Imaging System (SWIR vision).

### 2.9. Phototherapeutic Efficacy

For antitumor activity in vivo, the H22-tumor-bearing mice were randomly distributed between 5 groups (n = 5) for different treatments: (1) control group (saline); (2) saline with NIR laser only; (3) NV-p@BSA NPs (20 mg kg^−1^); (4) NV-PEG NPs (20 mg kg^−1^) with NIR laser irradiation; (5) NV-p@BSA NPs (20 mg kg^−1^) with NIR laser irradiation. For the laser treatment group, 808 nm light irradiation was conducted 6 h after injection at the power density of 1.0 W cm^−2^ for 10 min. During the experiment, tumor sizes were measured by a digital caliper, and body weights were recorded every 2 days. The in vivo treatment studies ended when the maximum tumor sizes of mice exceeded 20 mm. Tumor volume (V) was calculated by the following equation: V = (tumor length) × (tumor width)^2^/2. Relative tumor volume was calculated as V/V_0_ (V_0_ is the initial tumor volume of each individual mouse). At the end of treatments, the mice were euthanized, and the tumors were separated and weighed.

### 2.10. Toxicology Evaluation

Healthy Balb/c mice were intravenously injected with saline (control group) and NV-p@BSA NPs at a dose of 20 mg kg^−1^ (test group). After injection at different times (days 1, 7, and 28), mice were euthanized and then the blood was collected for biochemistry analysis. In addition, the major organs (heart, liver, spleen, lung, and kidney) were harvested and dissected to make paraffin sections for further H&E staining. The tissue slices were examined on an inverted fluorescence microscope system (Nikon Ti–S).

## 3. Results and Discussion

### 3.1. Synthesis and Characterization

**Figure 1:**
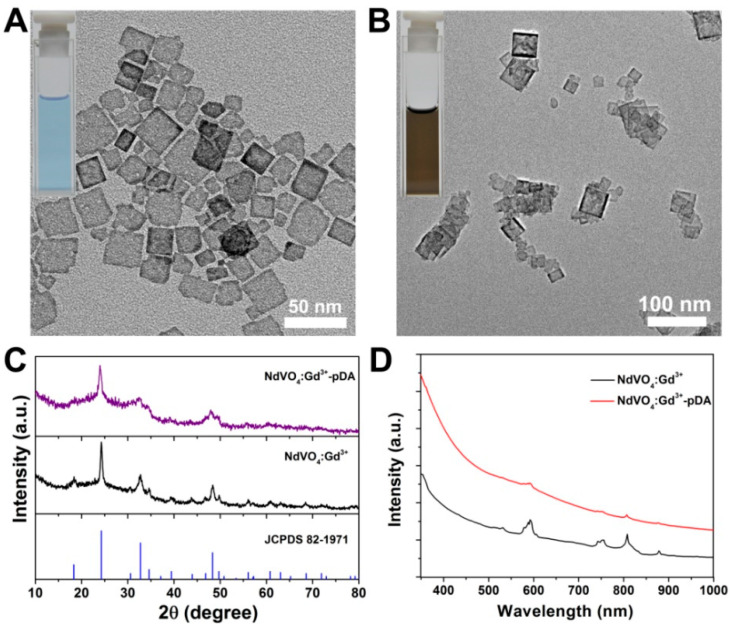
TEM images of NdVO_4_:Gd^3+^ (**A**) and NdVO_4_:Gd^3+^-pDA nanoplates (**B**); inset: the digital photos of NdVO_4_:Gd^3+^ in cyclohexane and NdVO_4_:Gd^3+^-pDA in DMSO, respectively. (**C**) XRD patterns of NdVO_4_:Gd^3+^ samples, NdVO_4_:Gd^3+^-pDA nanoplates, and the standard JCPDS card no. 82-1971. (**D**) Emission spectrum of NdVO_4_:Gd^3+^-pDA nanoplates under excitation of 808 nm laser light.

Oleic acid (OA)-stabilized NdVO_4_ NPs were synthesized via a facile hydrothermal method, and these nanomaterials were structurally uniform with a square-like plate shape (Figure S1). With the modified synthesis route, Gd^3+^-doped NdVO_4_ (NdVO_4_:Gd^3+^) NPs can also be prepared. The resulting NPs were analyzed using a variety of techniques including transmission electron microscope (TEM), X-ray diffraction (XRD), UV–VIS–NIR absorption, and photoluminescence spectroscopy. Typical TEM images revealed that the as-obtained NdVO_4_:Gd^3+^ NPs were of similar nanostructures as pristine NdVO_4_ NPs, with an average length of 24 ± 6 nm (Figure 1A). To make these NPs suitable for bioapplication, an effective means was utilized to transfer these hydrophobic nanoagents to water phase. Polydopamine (pDA) were grafted on the surface of OA-NdVO_4_:Gd^3+^ NPs through the polymerization of dopamine. As a capping agent, pDA layer had an evident influence on the color of NdVO_4_:Gd^3+^ NPs, which changed from blue to brown (Figure 1B and inset). Power XRD patterns revealed that both NdVO_4_:Gd^3+^ and NdVO_4_:Gd^3+^-pDA samples exhibited similar diffraction peaks in accordance with tetragonal NdVO_4_ nanocrystals (Figure 1C). It can be inferred that the 20% Gd^3+^ doping in NdVO_4_ nanocrystals did not affect their nanostructures. The UV–VIS–NIR absorption spectra showed that the as-obtained NdVO_4_:Gd^3+^ and NdVO_4_:Gd^3+^-pDA had the similar characteristic peak of Nd^3+^ ions at around 808 nm (Figure 1D), providing evidence that the resulting NdVO_4_:Gd^3+^-pDA NP solution exhibits a strong absorption at 808 nm. Furthermore, the emission spectrum of these NPs under the excitation of 808 nm laser was examined. As depicted in Figure S2, the strong emission band at 1060 nm for NdVO_4_:Gd^3+^-pDA NPs could be observed clearly, ascribed to the energy transfer from ^4^F_3/2_ level to ^4^I_11/2_ level of Nd^3+^ ions. This result implies that NdVO_4_:Gd^3+^-pDA NPs have a great potential as effective NR-II imaging agents [37].

To construct these NPs with good dispersity in aqueous solution, negatively charged BSA molecules were chosen as a model biological agent to conjugate on their surface. On the basis of the electrostatic interaction between BSA and amino groups of NdVO_4_:Gd^3+^-pDA NPs, BSA-modified NdVO_4_:Gd^3+^-pDA (NV-p@BSA) NPs were developed. As expected, the zeta-potential data showed the surface charge of NdVO_4_:Gd^3+^-pDA NPs (+30 mV) rapidly decreased to +6.7 mV after BSA coating (Figure S3). According to the thermogravimetric analysis (TGA) in Figure S4, the mass percent of the pDA layers in NdVO_4_:Gd^3+^-pDA nanoplates was evaluated as being around ≈14.5%. This modified strategy exhibited several advantages, including both coating layers (pDA and BSA) being biologically safe materials and the surface modification process being realized under a mild condition. Moreover, NV-p@BSA NPs were able to maintain their size after being dispersed in PBS solution for three days (Figure S5).

### 3.2. Assessment of In Vitro Photothermal Effect

**Figure 2:**
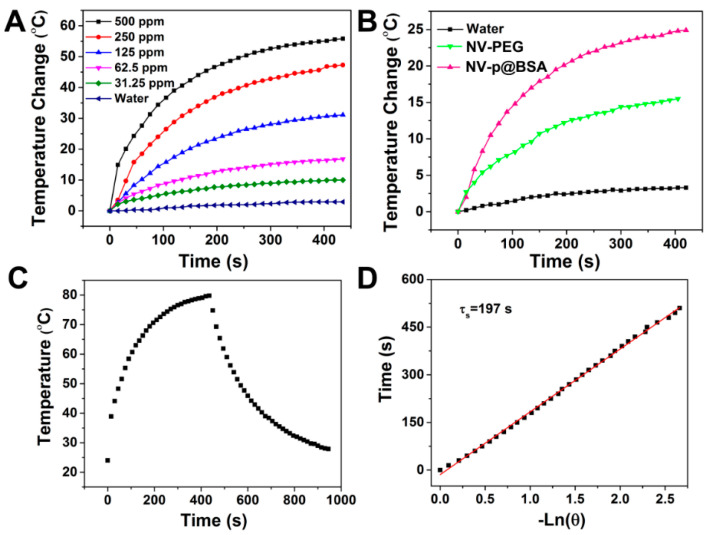
(**A**) Temperature elevation curves of NV-p@BSA solution with different concentrations when exposed to 808 nm NIR laser (1.0 W cm^−2^) for 5 min. (**B**) Temperature changes of NV-p@BSA and NV-PEG solution at the same concentration under 808 nm NIR laser excitation. (**C**) Temperature changes of 500 μg mL^−1^ NV-p@BSA aqueous solution over one laser on/off cycle of NIR laser irradiation. (**D**) Linear plot of time data versus −ln(*θ*) obtained from the cooling period of (**C**).

The possibility of NV-p@BSA NPs as photothermal agents was evaluated. The temperature changes of aqueous medium containing NV-p@BSA NPs during irradiation of 808 nm laser were monitored by a NIR camera. Figure 2A shows the photothermal responses of these NPs at concentrations varying from 31.25 to 500 μg mL^−1^ during the NIR irradiation process. It can be seen that the as-prepared NV-p@BSA samples displayed an apparent concentration-dependent photo-to-heat effect. After 5 min of laser irradiation, the temperature of NP solutions at a low concentration (125 μg mL^−1^) was able to increase from 25 °C to 55 °C, which is sufficient to destroy cancer cells. On the contrary, no significant temperature change was observed for deionized water under the same conditions, suggesting that the photothermal property originated from the as-prepared NPs. Moreover, to further study whether the pDA layer contributed to the photothermal efficiency of these NPs, PEGlyated-NdVO_4_:Gd^3+^ (NV-PEG) NPs were used as the control since the surface functionalization of PEG is a common strategy for phase transfer of OA-capped nanoparticles and PEG itself shows no signs of any photothermal properties. Compared with NV-PEG NPs, the resulting NV-p@BSA NPs exhibited superior light-to-heat conversion efficiency upon 808 nm laser excitation (Figure 2B). Therefore, it can be validated that the pDA layer can enhance the photothermal ability of NdVO_4_:Gd^3+^ NPs due to their own photothermal property, which is in line with previous reports [38,39,40].

Accordingly, to analyze the photothermal conversion efficiency of these NV-p@BSA NPs, we measured the temperature changes of NPs solution (500 μg mL^−1^) when irradiated with 808 nm laser (1.0 W cm^−2^) and subsequently recorded the cooling process after switching off the NIR laser. As depicted in Figure 2C, the temperature elevation during this process was monitored to achieve a heat generation–dissipation curve. On the basis of the reported method, the photothermal transduction efficiency of samples can be expressed as the following Equation (1):

(1) $$\eta =\frac{hS\left(T_{max}-T_{surr}\right)-Q_{dis}}{I\left(1-10^{-A_{808}}\right)}$$

where *T_max_* (K) is the equilibrium temperature, *T_surr_* (K) is ambient temperature of the surroundings, *Q_dis_* (W) is heat loss from light absorbed by the container, *A*_808_ is the absorbance of samples at 808 nm, h (W·cm^−2^·K^−1^) is heat transfer coefficient, and S (cm^2^) represents the surface area of the container.

According to this calculation, the heat conversion efficiency of NV-p@BSA NPs was estimated to be 31.7% (Figure 2D). The photothermal conversion properties of NV-p@BSA did not show significant changes after the three cycles, indicating their good thermal stability (Figure S6). All the results suggested that the as-prepared NV-p@BSA NPs could be a promising 808 nm laser-driven photothermal agent.

### 3.3. In Vitro Cytotoxicity Studies

**Figure 3:**
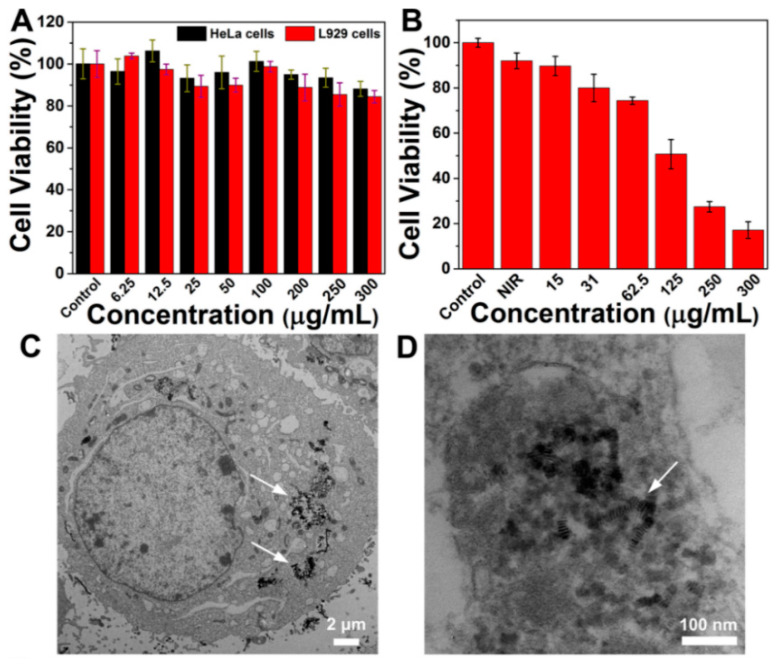
(**A**) In vitro cytotoxicity assays of NV-p@BSA NPs at various concentrations against HeLa and L929 cells. (**B**) Photo-induced cytotoxicity of NV-p@BSA samples plus the 808 nm laser irradiation (0.8 W cm^−2^, 5 min) towards HeLa cells. (**C**,**D**) TEM images of HeLa cells incubated with NV-p@BSA NPs for 4 h. The white arrows indicate the locations of NV-p@BSA NPs within the cells.

To examine the potential of these NPs as theranostic agents, in vitro dark cytotoxicity and NIR-induced photothermal cytotoxicity of NV-p@BSA NPs were evaluated using standard MTT assay. Two types of cells, normal cells (L929 cells) and tumor cells (HeLa cells), were used as model cells. After incubation with NV-p@BSA NPs in the concentration range of 0–300 μg mL^−1^ for 24 h, both cell viabilities were detected. As displayed in Figure 3A, no significant cytotoxicity in normal cells was observed in a high concentration of NPs (300 μg mL^−1^), as well as in HeLa cells, suggesting that these NPs can serve as a biocompatible nanoplatform for cancer therapy in living cells. In addition, the photothermal effect of NV-p@BSA NPs was further assessed at the cellular level. In absence of NPs, NIR laser irradiation alone caused negligible living cell death (Figure 3B). In contrast, a noticeable concentration-dependent cytotoxicity against the cancerous cells was observed in the exposure of HeLa cells to NV-p@BSA NPs for 4 h followed by laser irradiation. As we can see, the cells viability significantly decreased to only 17% when HeLa cells were treated with a dose of 300 μg mL^−1^ NPs plus laser irradiation. These data infer that these NPs could effectively absorb NIR light and generate increased temperatures sufficient to induce irreversible damage to cancer cells.

Subsequently, the cellular uptake behavior of NV-p@BSA NP was investigated on HeLa cells. As shown in Figure S7, the green fluorescence from FITC-labeled NV-p@BSA NP and the blue fluorescence from DAPI-labeled cell nucleus exhibited effective overlay, verifying that NV-p@BSA NP could be taken up by HeLa cells. Meanwhile, biological TEM images of the treated cells were used to monitor their cellular location. As shown in Figure 3C,D, NV-p@BSA NPs were found to be effectively phagocytized by HeLa cells and were mainly located inside the endosomes. These data provide insight into the effective cellular uptake of NV-p@BSA NPs by tumor cells, which makes it possible for these NPs to eradicate cancer cells under the irradiation of an 808 nm laser.

### 3.4. In Vitro and In Vivo Multimodal Imaging

**Figure 4:**
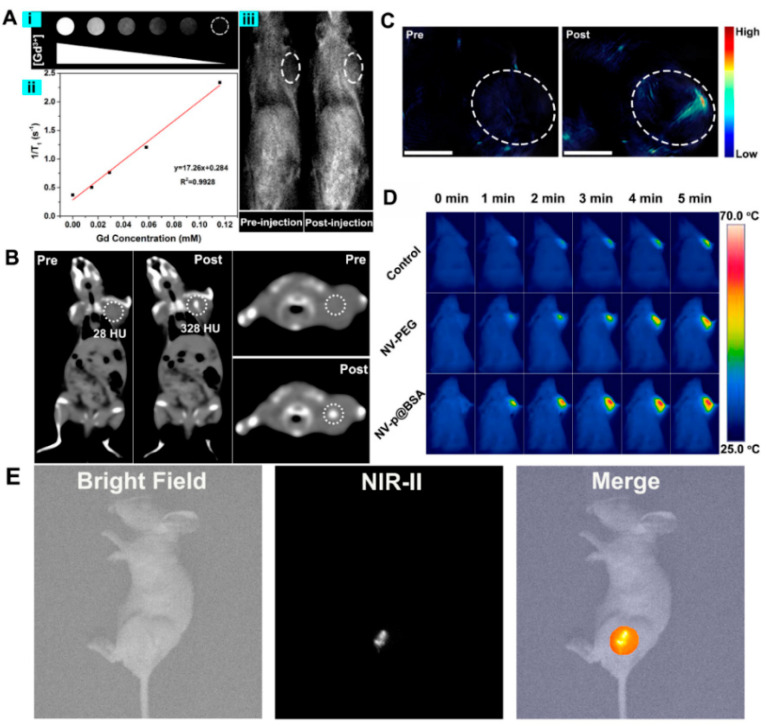
(**A**) Representative *T*_1_-weighed MR contrast effects of NV-p@BSA NPs at different concentrations (i); graphs of relaxation rates R_1_ versus different concentrations of NPs (ii); in vivo MR images of tumor-bearing mice before and after injection of NV-p@BSA NPs (iii). (**B**) In vivo CT images of tumor-bearing mice before and after injection of NV-p@BSA NPs. The white circles indicate the tumor regions. (**C**) Representative PA images at the tumor region before and after injection of NV-p@BSA NPs. Representative thermal images (**D**) and NIR-II fluorescence images (**E**) of mice subjected to 808 nm laser irradiation (1.0 W cm^−2^).

Versatile nanoplatforms with high contrast ability are urgently needed for guiding the therapeutic treatment in avoidance of damaging the surrounding healthy tissues. In this study, the in vivo multimodal imaging performance of as-prepared NV-p@BSA NPs was evaluated on a H22 xenograft model, including *T*_1_-weighted MR, CT, PA, IR, and NIR-II imaging. Firstly, the in vitro MR nature of these Gd^3+^-doped NPs was confirmed by measuring *T*_1_ value as a function of their concentrations. Figure 4A demonstrates the *T*_1_ MR signals were enhanced gradually with the increasing concentration of these solutions, which exhibited a noticeable brightening effect. The trend was well fitted by a linear line within the tested range of Gd^3+^ concentrations. The *T*_1_ relaxivity coefficient value was estimated to be 1.726 Mm^−1^ s^−1^. To investigate the in vivo MR imaging of NV-p@BSA NPs, MR images of tumor-bearing mice before and after the injection of NPs (100 μg mL^−1^) were acquired. In the post-injection image, an MRI signal enhancement on the tumor site was observed.

X-ray computed tomographic (CT) imaging is proven with high resolution and deep penetration for clinical diagnoses. Figure 4B presents the in vivo CT images acquired before and after intratumoral injection of NV-p@BSA NPs (100 mL, 5 mg mL^−1^) into tumor-bearing mice. Compared with the intrinsic tumor image pre-injection, the injected tumor area exhibited a brighter contrast effect, suggesting a significant increase in the CT signal. To demonstrate the feasibility of NV-p@BSA NPs for PA imaging, in vivo three-dimensional PA images of the tumor region were also conducted for NP-injected mice. For those treated with NV-p@BSA NPs, obvious PA amplitude for mice was revealed in comparison to the untreated tumor (Figure 4C). As shown in Figure 4D, the temperature of the tumor site in normal saline injection mice was not significantly increased in the control group, which confirmed that such a near infrared light irradiation condition had no additional damage to the tumor site. The surface temperature of the tumor increased rapidly after in situ injection of the same concentration of NV-PEG and NV-p@BSA, while the temperature of the latter material was significantly higher than that of the former under the same light irradiation conditions, indicating that the NV-p@BSA performed a higher heat production ability for tumor thermal ablation. The NIR emission peak of Nd^3+^ ions can be derived from radiative transitions of ^4^F_3/2_→^4^I_11/2_ (1060 nm), suggesting NV-p@BSA could be served as NIR-II fluorescence with favorable photostability. To evaluate the NIR-II fluorescence imaging feasibility of NV-p@BSA NPs in vivo, the whole-body imaging of tumor-bearing mice within the NIR-II region was conducted for NP intratumoral injection. For those treated with NV-p@BSA NPs, a strong NIR-II fluorescence signal for mice was detected in the tumor region (Figure 4E). Taken together, all these above data support that the NV-p@BSA NPs could be promising agents for synchronous MR/CT/PA/IR/NIR-II imaging, which may be useful for cancer diagnosis, owing to the excellent depth of visualization in tumor tissues.

### 3.5. In Vivo Photothermal Effect

The in vivo photothermal effect of as-obtained NV-p@BSA NPs were investigated via infrared thermal imaging and photothermal ablation of the tumor. Before in vivo NIR-light-triggered treatment, 200 μL of saline, NV-PEG, and NV-p@BSA solution was injected into the tumor-bearing mice. Under 808 nm laser irradiation, the cutaneous temperature of the tumor region was recorded by an infrared thermo-camera as a function of time. As described in Figure 4D, it produced a well localized hyperthermal and accelerated temperature response inside the tissue in the NV-PEG-NP-treated group, showing that the temperature increased to 50 °C. In comparison, the tumor temperature reached up 65 °C in the NV-p@BSA-treated mice. This implies that NV-p@BSA NPs exhibit more potent photothermal performance over NV-PEG NPs. Nevertheless, only inapparent thermal absorption by tissue was observed in NIR-laser-treated mice, suggesting the laser irradiation is safe for surrounding healthy tissues but enough to activate these NPs to induce photodamage to the tumor site. On the basis of these infrared thermal images, we found the heating spot was only limited to the tumor region, suggesting that the thermal damage could be constructed in the area of interest and that the surrounding normal cells were weakly affected.

**Figure 5:**
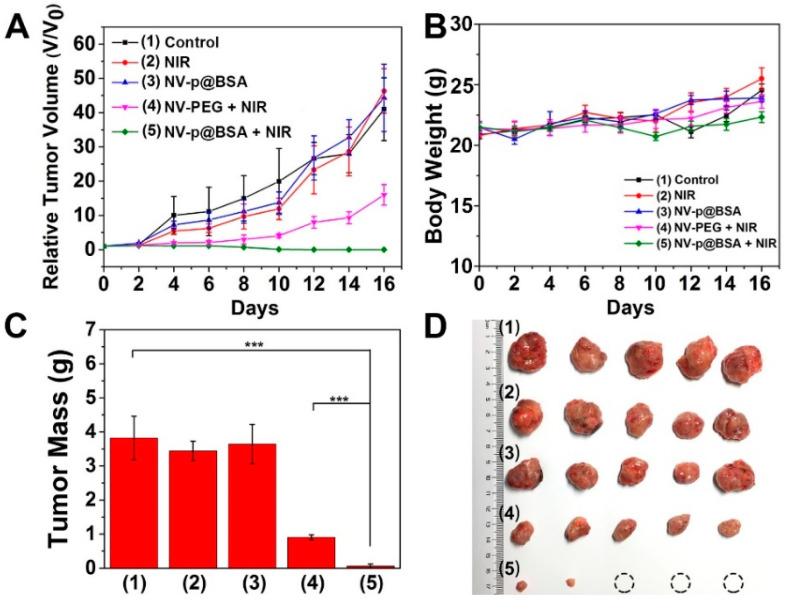
(**A**) Relative tumor volume changes and (**B**) corresponding body weight changes of tumor-bearing Balb/c mice after different treatment processes, as indicated. (**C**) Average weights of tumors collected from mice in each treatment group. (**D**) Photos of representative excised tumor tissues from different groups of mice at the end of treatments. *p*-values: *** *p* < 0.001.

Motivated by the outstanding in vitro photothermal performance and low cytotoxicity, the in vivo antitumor activity of this NV-p@BSA nanosystem was further investigated in tumor-bearing Balb/c mice that were inoculated subcutaneously with H22 hepatocellular carcinoma cells. These mice were randomly assigned into five groups and treated with an intratumoral injection of different samples, including saline, NV-PEG NPs, or NV-p@BSA NPs. In NIR-laser-treated groups, an 808 nm laser was focused on the tumor site of mice for 5 min (1.0 W cm^−2^) after injection. The tumor growth was monitored within 28 days following the treatment. As displayed in Figure 5A, there was no significant difference in final tumor size for saline-, saline + NIR-, or NV-p@BSA-treated groups, indicating that these treatments had little effect on tumor inhibition. Once laser irradiation was applied, the tumor growth of mice treated with NV-PEG NPs was efficiently delayed. As expected, the tumor growth was greatly suppressed in the NV-p@BSA + NIR treated group, implying their pronounced photothermal performance (Figure 5B,C). These results also suggest that tHE pDA modification of these NPs further enhances the therapeutic efficacy when compared with NV-PEG NPs without the pDA layer, enabling them to be more effective in tumor photoablation.

### 3.6. In Vivo Biosafety Investigation

**Figure 6:**
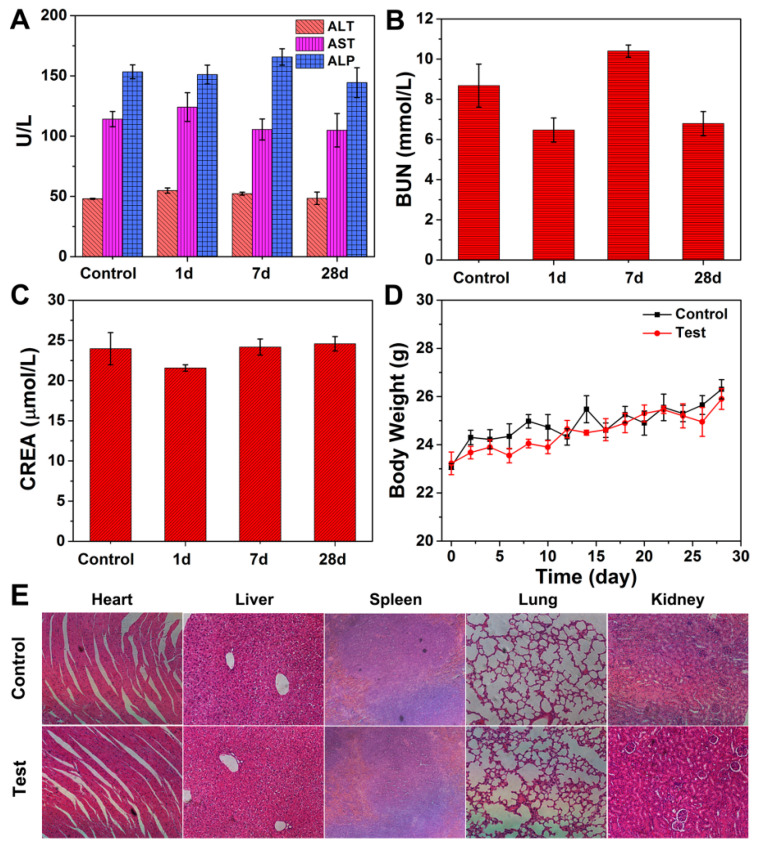
(**A**–**C**) Blood serum biochemistry data of healthy Balb/c mice with intravenous injection of NV-p@BSA NPs. The data were collected at different time points (1st, 7th, and 28th days post-injection). (**D**) Body weight change of Balb/c mice treated with NV-p@BSA NPs within 28 days compared to the control group without any injection. (**E**) H&E-stained histological slices from mice receiving no injection (control) or mice injected with NV-p@BSA NPs (test) at the 28th day post-injection.

To assess the potential long-term toxicity of intratumorally injected NV-p@BSA NPs after photothermal ablation, a simulated in vivo toxicity assessment was performed by injecting NPs (20 mg kg^−^^1^) into healthy Balb/c mice via the tail vein. This investigation could be achieved by monitoring blood biochemical index, body weight gain, and histological examinations. At the examined time points post-injection, the blood from NP-treated mice was collected and some important indicators were analyzed, including alanine aminotransferase (ALT), alkaline phosphatase (ALP), and aspartate aminotransferase (AST) for hepatic function, as well as blood urea (BUN) and serum creatinine (CREA) for renal function. As shown in Figure 6A–C, all these parameters were at a normal level and showed no visible differences compared with control group injected with saline. These biochemical data imply that no obvious abnormalities were associated with the treatment. In addition, all mice in the test group displayed similar changing profiles of body weight gain as the control group (Figure 6D), demonstrating that there were no acute side effects caused by our nanotherapeutic agent. To further assess the in vivo biocompatibility of these NPs, major organs of these injected mice were harvested on the 28th day post-injection and sliced for histology analysis. Hematoxylin and eosin (H&E)-stained sections suggest that no apparent lesion such as inflammatory response and necrocytosis was observed in the tissue sections (Figure 6E), further manifesting the low toxicity of the NV-p@BSA NPs during the experiment.

Overall, these preliminary results show that NV-p@BSA NPs had low side effects at the tested dose in vivo, suggesting their potential as a therapeutic nanoplatform for intravenous photothermal treatments. As a multifunctional nanoagent, NV-p@BSA NPs have potential application for multiple imaging-guided photothermal therapies in theranostic nanomedicine, although further in vivo toxicity studies and functional modification are still needed to be carried out.

## 4. Conclusions

In summary, this work reports a multifunctional NV-p@BSA nanoplatform for multimodal-imaging-guided photothermal therapy in vivo, owing to their excellent photothermal conversion capability and functional compositions. The NV-p@BSA nanosystem shows little appreciable dark toxicity and can serve as an efficient photothermal agent with irradiation time and dosage dependence when exposed to external NIR laser. With the effective cellular uptake by cancer cells, these NPs are proven to induce severe photo-cytotoxicity in vitro and exert significant tumor ablation post-injection in vivo. Remarkably, these NPs are capable of in vivo MR/CT/PA multimodal imaging. Therefore, we believe that these NV-p@BSA NPs hold great potential as theranostic agents for accurate diagnosis and efficient photothermal ablation of tumors.

## Data Availability

The results of this paper are entirely documented in the figures of this paper.

## Supplementary Materials

The following supporting information can be downloaded at: https://www.mdpi.com/article/10.3390/pharmaceutics14061217/s1, Figure S1: TEM image of as-obtained NdVO_4_ nanoplates. Figure S2: NIR-II emission spectrum of as-obtained NdVO_4_:Gd^3+^-pDA solution. Figure S3: Surface charges of NdVO_4_:Gd^3+^-pDA (1) and NV-p@BSA (2) aqueous solution. Figure S4: Typical thermal gravimetric analysis curve of NdVO_4_:Gd^3+^ and NdVO_4_:Gd^3+^-pDA nanoplates. Figure S5: The particle size of NV-p@BSA NPs in PBS solution over 72 h. Figure S6: Temperature increases by NV-p@BSA over three on/off cycles of 808 nm irradiation. Figure S7: The confocal microscope images of HeLa cells after being cultured with NV-p@BSA NPs for 24 h.
