# Supplementary material for: Surface-Functionalized NdVO4:Gd3+ Nanoplates as Active Agents for Near-Infrared-Light-Triggered and Multimodal-Imaging-Guided Photothermal Therapy
Source: Pharmaceutics. 2022 Jun 8;14(6):1217. doi: 10.3390/pharmaceutics14061217 (PMC9230566; doi:10.3390/pharmaceutics14061217)
Supplement: Supplementary file 1 [file pharmaceutics-14-01217-s001.zip › pharmaceutics-1692567-supplementary.pdf]

Surface-Functionalized $NdVO_4:Gd^{3+}$ Nanoplates as Active Agents for Near-Infrared-Light-Triggered and Multimodal-Imaging-Guided Photothermal Therapy

Kerong Deng [1,2,3,†], Donglian Liu [1,†], Ziyan Wang [4,†], Zhaoru Zhou [2], Qianyi Chen [2], Jiamin Luo [2], Yaru Zhang [1,2,*], Zhiyao Hou [1,2,*] and Jun Lin [1,3,*]

[1] The Sixth Affiliated Hospital of Guangzhou Medical University, Qingyuan People's Hospital, Qingyuan 511518, China; dengkr26@gmail.com (K.D.); ldl00987@163.com (D.L.)
[2] Guangzhou Municipal and Guangdong Provincial Key Labrotory of Protein Modification and Degradation, School of Basic Medical Sciences, Guangzhou Medical University, Guangzhou 511436, China; 2019218158@stu.gzhmu.edu.cn (Z.Z.); 2019218157@stu.gzhmu.edu.cn (Q.C.); jiaminluo7@stu.gzhmu.edu.cn (J.L.)
[3] State Key Laboratory of Rare Earth Resource Utilization, Changchun Institute of Applied Chemistry, Chinese Academy of Sciences, Changchun 130022, China
[4] The First Clinical School of Guangzhou Medical University, Guangzhou Medical University, Guangzhou 510120, China; wangzy2018@stu.gzhmu.edu.cn
* Correspondence: zyr@gzhmu.edu.cn (Y.Z.); zyhou@gzhmu.edu.cn (Z.H.); jlin@ciac.ac.cn (J.L.)
† These authors contributed equally to this work.

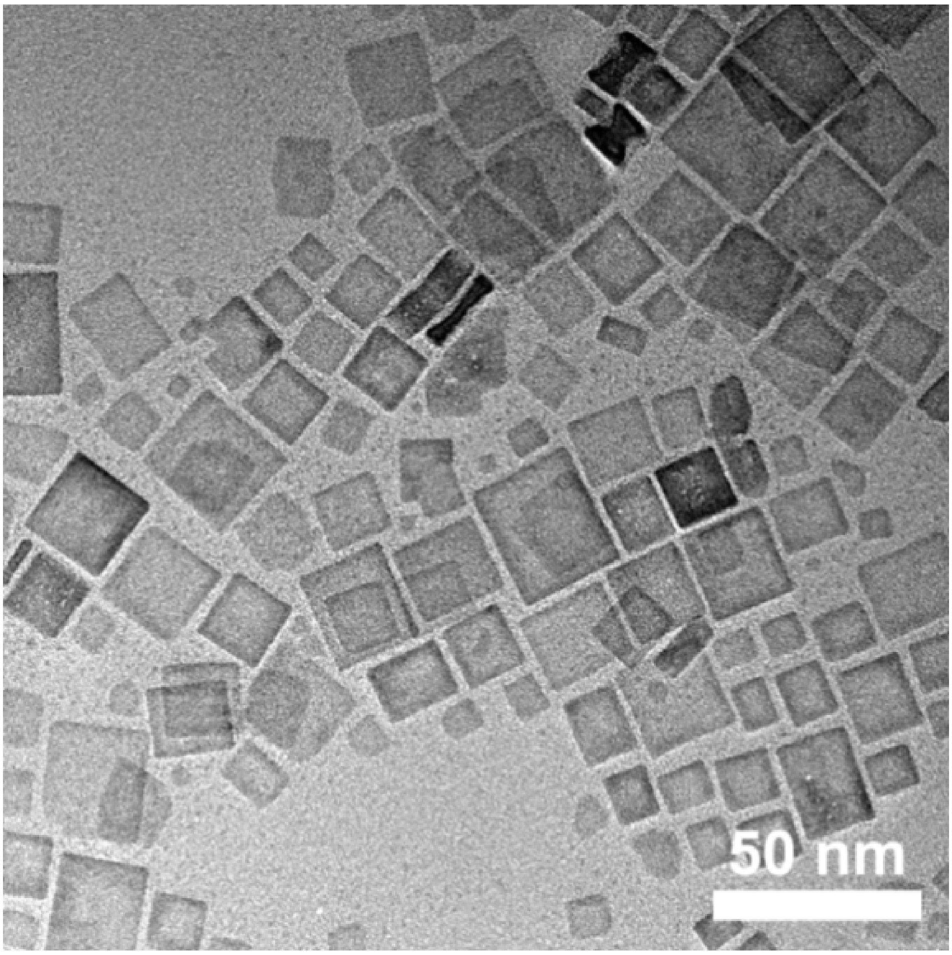

Figure S1. TEM image of as-obtained $NdVO_4$ nanoplates.

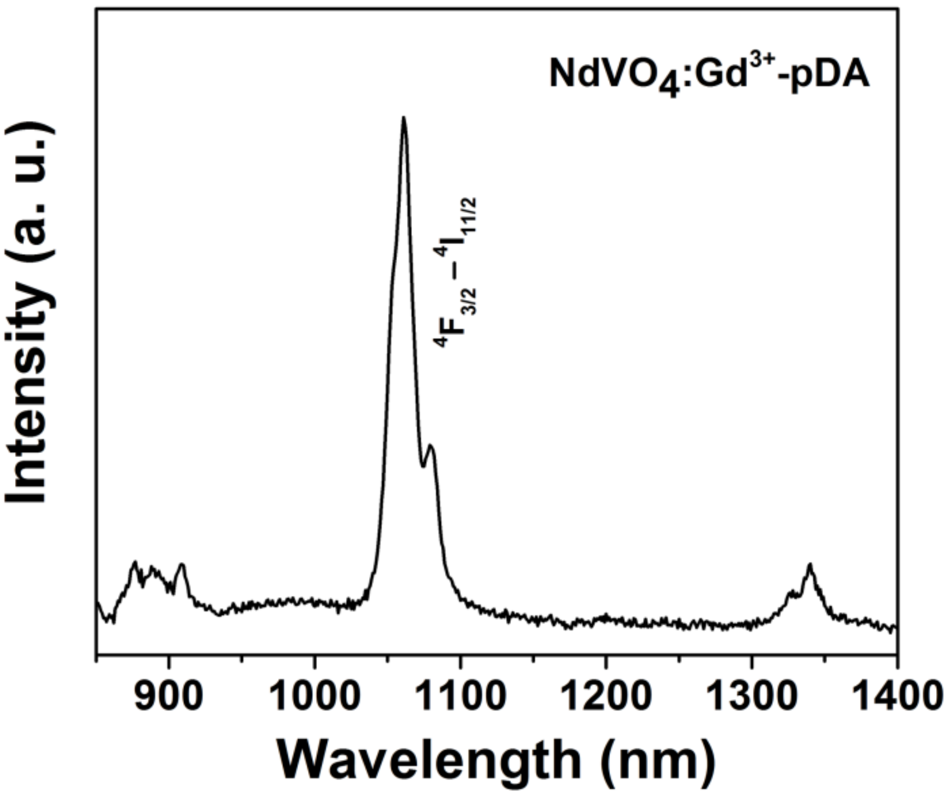

Figure S2. NIR-II emission spectrum of as-obtained $NdVO_4:Gd^{3+}$-pDA solution.

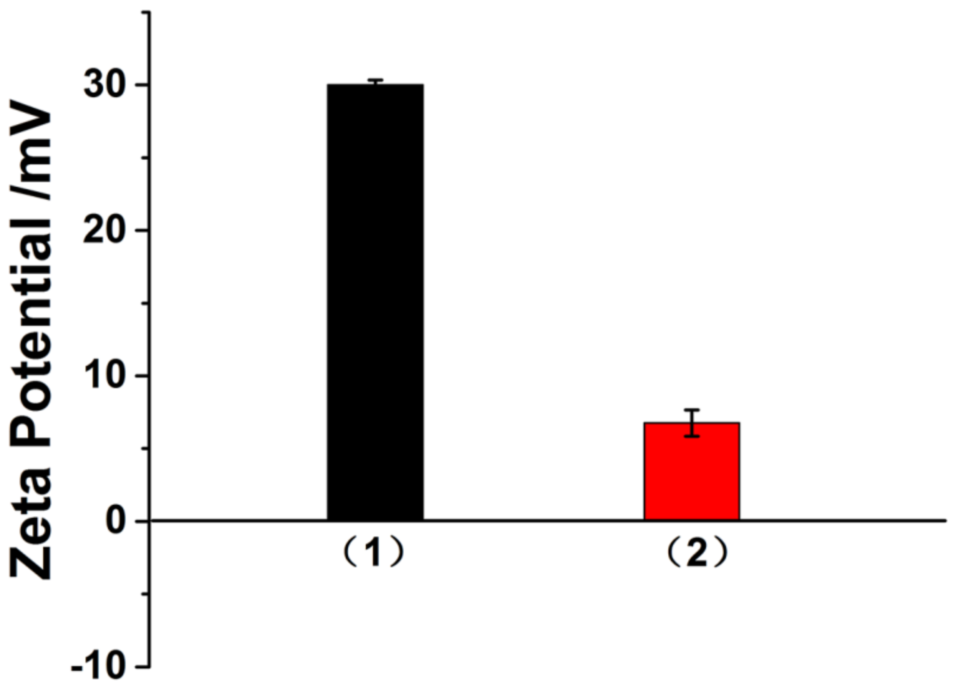

Figure S3. Surface charges of $NdVO_4$:Gd^{3+}-pDA (1) and NV-p@BSA (2) aqueous solution, respectively.

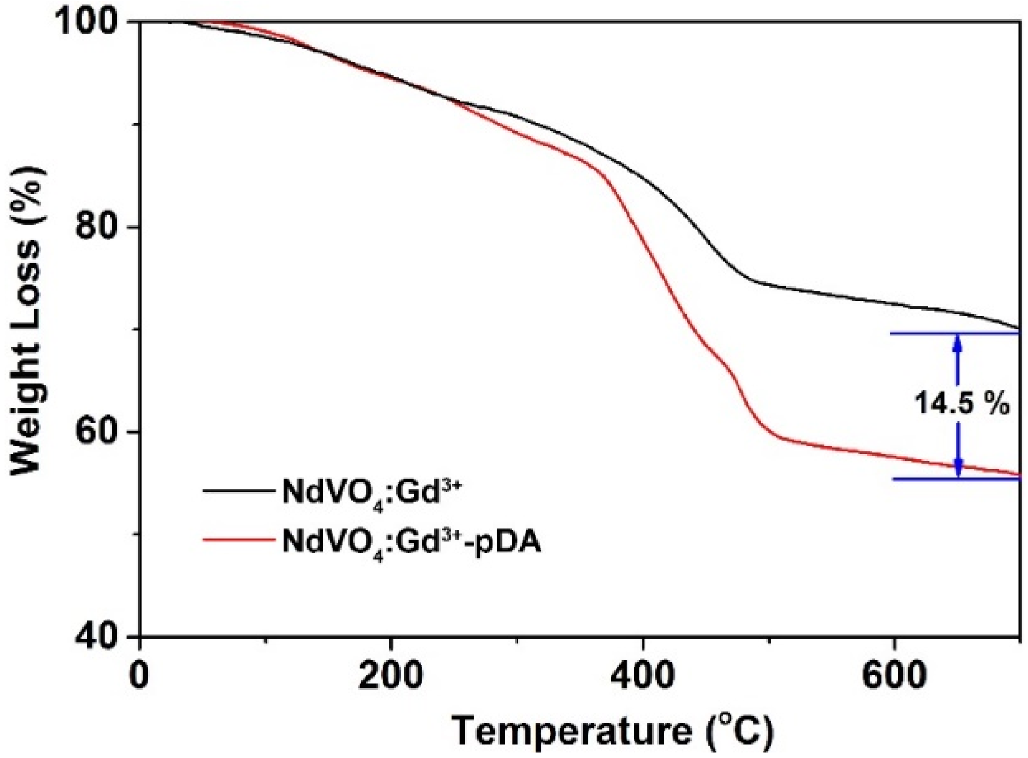

Figure S4. Typical thermal gravimetric analysis curve of $NdVO_4:Gd^{3+}$ (black line) $NdVO_4:Gd^{3+}$-pDA (red line) nanoplates, respectively.

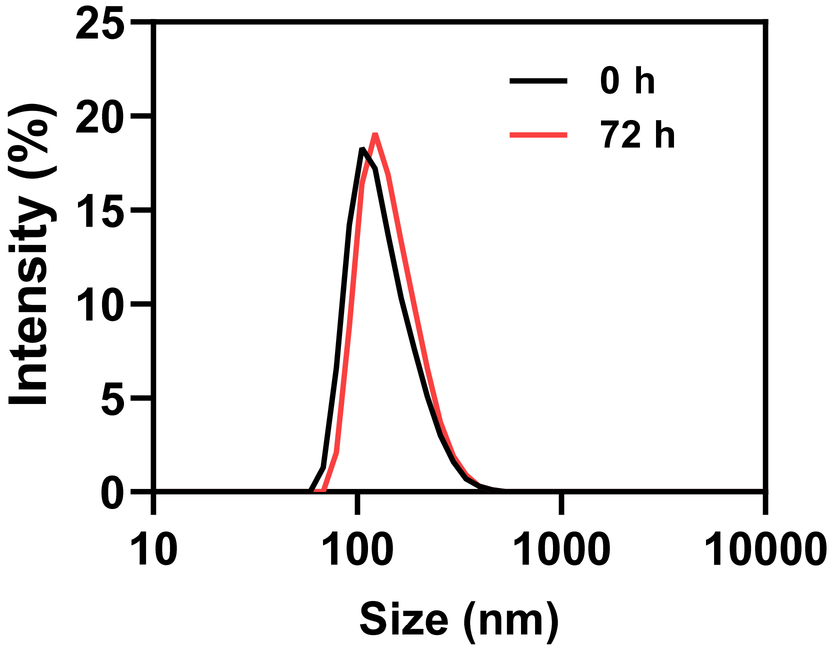

Figure S5. The particle size of NV-p@BSA NPs in PBS solution over 72 h.

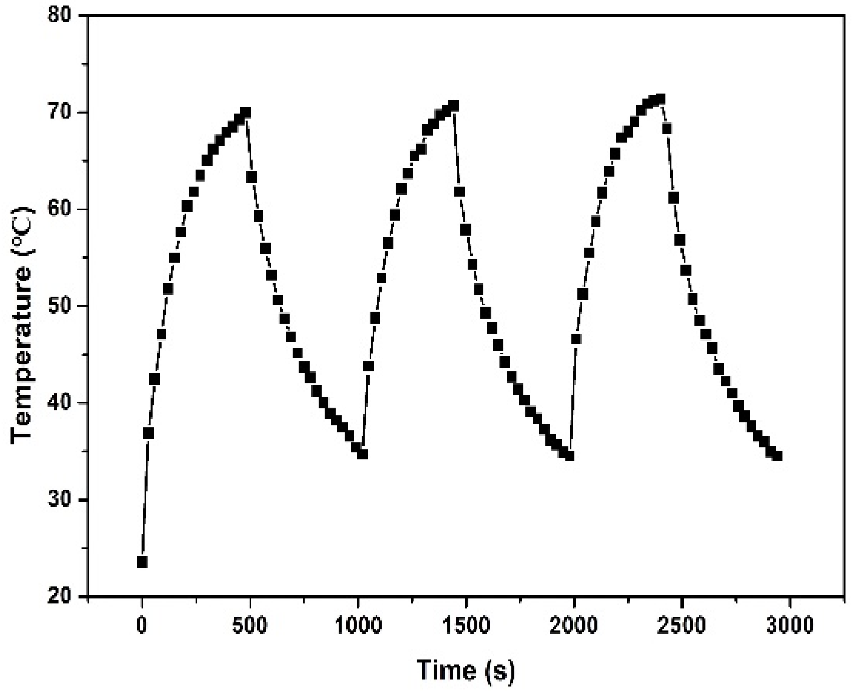

Figure S6. Temperature increases by NV-p@BSA over three on/off cycles of 808-nm irradiation.

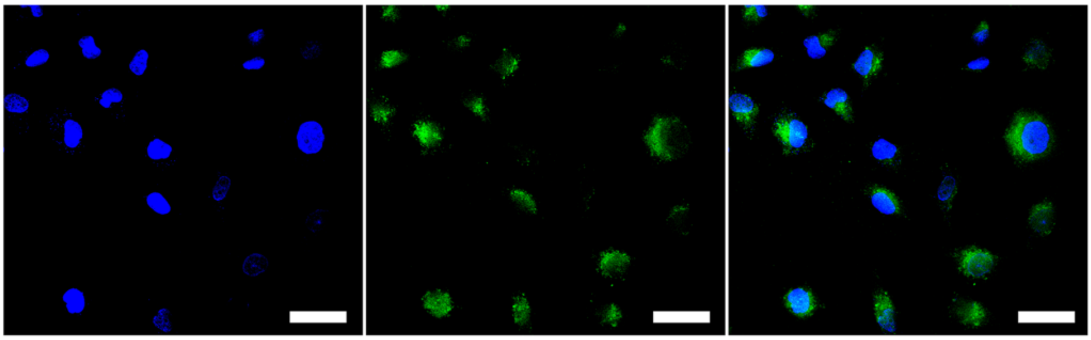

Figure S7. The confocal microscope images of HeLa cells after cultured with NV-p@BSA NPs for 24 h. Blue and green fluorescence in those images represent DAPI-stained cell nuclei and FITC-labeled NV-p@BSA NPs, respectively. All scale bars were 50 μm.
